# Effects of systemic ozone administration on the fresh extraction sockets healing: a histomorphometric and immunohistochemical study in rats

**DOI:** 10.1590/1678-7757-2023-0412

**Published:** 2024-05-13

**Authors:** Erton Massamitsu MIYASAWA, Edilson ERVOLINO, Jânderson de Medeiros CARDOSO, Leticia Helena THEODORO, Glauco Rodrigues Carmo SILVEIRA, Rafael Scaf de MOLON, Liran LEVIN, Valdir Gouveia GARCIA, Luis Eduardo Marques PADOVAN

**Affiliations:** 1 ILAPEO Curitiba PR Brasil Instituto Latino Americano de Pesquisa e Ensino Odontológico (ILAPEO), Curitiba, PR, Brasil.; 2 UNESP Faculdade de Odontologia de Araçatuba Grupo de Pesquisa e Estudo com Laser em Odontologia Araçatuba São Paulo Brasil Universidade Estadual Paulista - UNESP, Faculdade de Odontologia de Araçatuba, Grupo de Pesquisa e Estudo com Laser em Odontologia, Araçatuba, São Paulo, Brasil.; 3 UNESP Faculdade de Odontologia de Araçatuba Departamento de Ciências Básicas Araçatuba São Paulo Brasil Universidade Estadual Paulista - UNESP, Faculdade de Odontologia de Araçatuba, Departamento de Ciências Básicas, Araçatuba, São Paulo, Brasil.; 4 UNESP Faculdade de Odontologia de Araçatuba Departamento de Diagnostico e Cirurgia Araçatuba São Paulo Brasil Universidade Estadual Paulista - UNESP, Faculdade de Odontologia de Araçatuba, Departamento de Diagnostico e Cirurgia, Araçatuba, São Paulo, Brasil.; 5 University of Alberta Faculty of Medicine and Dentistry Canada University of Alberta, Faculty of Medicine and Dentistry, Canada.

**Keywords:** Ozone therapy, Bone healing, Animal research, Rats

## Abstract

Studies have highlighted numerous benefits of ozone therapy in the field of medicine and dentistry, including its antimicrobial efficacy against various pathogenic microorganisms, its ability to modulate the immune system effectively, reduce inflammation, prevent hypoxia, and support tissue regeneration. However, its effects on dental extraction healing remain to be elucidated.

**Objective:**

Therefore, this study aimed to evaluate the effects of systemically administered ozone (O_3_) at different doses in the healing of dental extraction sockets in rats.

**Methodology:**

To this end, 72 Wistar rats were randomly divided into four groups after extraction of the right upper central incisor: Group C – control, no systemic treatment; Group OZ0.3 – animals received a single dose of 0.3 mg/kg O_3_; Group OZ0.7 – a single dose of 0.7 mg/kg O_3_; and Group OZ1.0 – a single dose of 1.0 mg/kg O_3_, intraperitoneally. In total, six animals from each group were euthanized at 7, 14, and 21 days after the commencement of treatment. Bone samples were harvested and further analyzed by descriptive histology, histomorphometry, and immunohistochemistry for osteocalcin (OCN) and tartrate-resistant acid phosphatase (TRAP) protein expression.

**Results:**

All applied doses of O_3_ were shown to increase the percentage of bone tissue (PBT) after 21 days compared to group C. After 14 days, the OZ0.7 and OZ1.0 groups showed significantly higher PBT when compared to group C. The OZ1.0 group presented the most beneficial results regarding PBT among groups, which denotes a dose-dependent response. OCN immunostaining was higher in all groups at 21 days. However, after seven and 14 days, the OZ1.0 group showed a significant increase in OCN immunostaining compared to C group. No differences in TRAP+ osteoclasts were found between groups and time points.

**Conclusion:**

Therefore, O_3_ therapy at higher doses might be beneficial for bone repair of the alveolar socket following tooth extraction.

## Introduction

Ozone is a natural compound present in the stratosphere that plays an important role in retaining ultraviolet energy emitted by the sun, thus contributing not only to control the thermal conditions of the stratosphere but also human life.^1^ From its discovery in the mid-1840s by the German chemist Christian Friedrich Schönbein^2^ and with the advent of the first ozone generator developed by Werner von Siemens in 1857,^3^ new horizons have been opened up for its use in both medicine and dentistry.^2,4,5^

Medical ozone is a gaseous mixture composed of 95 to 99.95% oxygen and 0.05 to 5% pure ozone, which can be found in gas or liquid forms (water or oil)^2,6^ and can be applied topically, infiltratively, or systemically. Ozone presents greater solubility in water and is 1.6 times denser than pure oxygen, with a half-life of 40 min at 20°C and degrades quickly into pure oxygen.^3^ Numerous studies have documented its effects, highlighting its bioenergetics, analgesic, anti-hypoxia, and lethal effects on bacteria, protozoa, fungi, and viruses,^7,8^ as well as on the oxygenation of tumors^9^ and in the therapy of HIV-AIDS^10^ and COVID-19.^11^

A previous study had demonstrated the ability of ozone to modulate the cellular antioxidant system and the inflammatory system, in addition to regulating oxygen metabolism in red blood cells, making a higher rate of oxygen available to tissues.^4^ The feasible benefits of O_3_ in dentistry and medicine are commonly attributed to its antimicrobial, disinfectant, and healing properties.^12^

The inclusion of ozone as a treatment focus in our study stems from previous research showcasing its positive impacts in medical sciences. In dentistry, ozone therapy is widely employed in endodontics, orthodontics, periodontics (specifically for treating gingivitis and periodontitis, as adjuvants),^13^ maxillofacial surgery^14^ for addressing mucosal lesions, treating oral lichen planus, managing osteonecrosis of the jaw, maintenance of bone mass,^15^ and in conservative dentistry (for remineralization, depigmentation, and desensitization of teeth).^8,15-18^ Additionally, ozone is utilized in the prevention and treatment of dental caries,^19^ disinfection of dentin tubules,^20,21^ and in implant dentistry.^22^ Those studies have consistently demonstrated favorable outcomes with the use of ozone therapy. Furthermore, recent clinical studies have explored the efficacy of ozone in treating periodontitis, revealing encouraging results.^23-26^ The effectiveness of ozone therapy in reducing microbial burden and enhancing immune system capabilities associated with minor side effects makes it a viable option for application in clinical studies.^23-27^

There are some advantages of using the ozone therapy in dentistry. The most reported beneficial effects are its antimicrobial efficacy against many pathogenic microorganisms,^25,28^ its effectiveness in modulating the immune system, reducing inflammation, preventing hypoxia, its biosynthetic effect, and supporting tissue regeneration.^25^ Besides, most of the studies showed no adverse side effects when using the different route of administration and dosages of ozone for dental application. Moreover, the possibility to use ozone therapy as hydrogel appears to be beneficial for treatment of periodontitis and other conditions, which can be performed at the dental office or at home.^25^ On the other hand, its use presents limitations. Some potential side effects that may arise include coughing, nausea, vomiting, headaches, inflammation of the nasal passages, and respiratory tract irritation. The most observed side effects include excessive tearing, irritation of the upper respiratory tract, rhinitis, coughing, headaches, occasional nausea, and vomiting. However, exposure to ozone at a concentration of 0.05 parts per million (ppm) for 8 hours have been shown to induce no adverse effects. The highest level of ozone encountered during dental procedures is 0.01 ppm.^29,30^ Thus, the ozone at 0.05 ppm does not seem to cause any side effect for its clinical use.

Despite all these advantages and the widespread application of ozone in dentistry, the role of ozone in the healing of fresh extraction sockets remains to be clarified.^6,12,31^ Therefore, this study aimed to evaluate the effects of ozone therapy on bone repair of post-extraction dental sockets in clinically healthy rats. The study hypothesis was that ozone therapy might benefit bone repair in a dose dependent manner.

## Methodology

### Animals

This study included 72 male Wistar rats (*Rattus norvegicus albinus*) aged 3 months old with mean body weight of 250–300g. The animals were housed in propylene cages (four animals per cage), with controlled temperature (21°C) and humidity (65–70%), and a 12-hour light-dark cycle. Animals consumed standard rat chow (Labina/Purina, Ribeirão Preto, Brazil) and received water *ad libitum*. The Research Ethics Committee for the use of animals approved this protocol (Proc. #123/2021), and the experimental study design followed all the Animal Research: Reporting of In Vivo Experiments (ARRIVE) guidelines.^32^

### Sample size calculation

The GPower^®^ software was used to calculate the sample size. Considering a 0.05 alpha (type I error) and 0.80 beta (type II error) with a medium effect size (ES=0.25), the number of study groups was calculated as a total of 64 animals. Bearing in mind possible complications and sample losses, a margin of 15% was included, resulting in a total number of 72 animals.

### Tooth extraction

All animals were previously weighed and subjected to general anesthesia via intramuscular injection of a combination of ketamine hydrochloride (80 mg/Kg, Francotar, Virbac, SP, Brazil) and xylazine hydrochloride (10 mg/Kg, Coopazine, Coopers Brasil Ltda, Cotia, SP, Brazil). After antisepsis of the surgical area, the flap was raised, the tooth was carefully dislocated, and the extractions of the upper right central incisor of each animal were performed, as previously described.^33^ Then, the soft tissue was sutured with polygalactin 910 thread (4-0, Johnson & Johnson, São José dos Campos, SP, Brazil). After suturing, all animals received a single dose of 0.2 ml of antibiotic (Pentabiotic C Veterinário Reforçado Wyeth S.A., Indústrias Farmacêuticas, São Bernardo do Campo, SP, Brazil) and 5mg/kg of analgesic (Tramadol^®^, Janssen-Cilag Farmacêutica Ltda, São Paulo, SP, Brazil) intramuscularly.

### Study design

Rats were randomly distributed into four groups (n=18) using a table generated by the website Randomization.com (http://www.randomization.com), as follows: C (Control) – animals did not receive any treatment; OZ0.3 – the animals received an intraperitoneal injection of ozone in a dose of 0.3 mg/kg with a concentration of 15 μg/ml of O_3_; OZ0.7 – the animals received an intraperitoneal injection of ozone in a dose of 0.7 mg/kg with a concentration of 35 μg/ml of O_3_; and OZ1.0 – the animals received an intraperitoneal injection of ozone in a dose of 1.0 mg/kg with a concentration of 50 μg/ml of O_3_. The Philozon Medplus V^®^ ozone generator (Philozon, Balneário Camboriu, SC, Brazil) was used, which automatically regulates oxygen flow and allows adjusting concentrations from 5 to 60 μg/ml. Intraperitoneal injections were performed by a single and experienced examiner. Ozone dose quantities were selected following a study by Erdemci, et al.^12^ (2014).

### Histological processing and histopathological analysis

In total, six animals from each group were euthanized via anesthetic overdose (Tiopental^®^, 150 mg/kg, Cristália, Itapira, São Paulo, Brazil) after seven, 14, and 21 postoperative days. The jaws containing the tooth extraction site were carefully dissected and kept in 4% formaldehyde in 0.1 M phosphate buffer (pH 7.4) for 48 hours. After fixation, the samples were subjected to demineralization in 10% ethylenediaminetetraacetic acid (EDTA; Sigma-Aldrich) in PBS for 60 days. Once demineralization was completed, the samples were dehydrated in ethanol, cleared in xylene, and impregnated and embedded in paraffin, as described elsewhere.^34,35^ The microtome cut was performed following a longitudinal cutting plane in relation to the dental socket, and serial cuts involving the central portion of the tooth extraction site were made with 5 µm thickness and collected on silanized glass slides.

### Histomorphometric analysis

**Figure 1:**
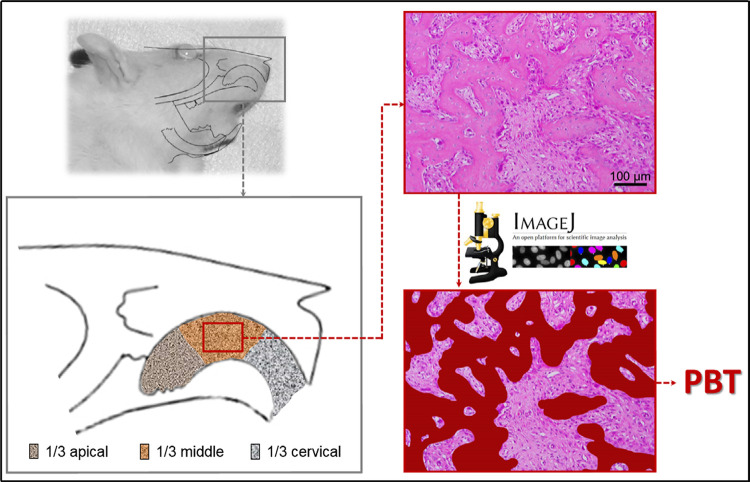
Representative images of the region of interest (ROI) for histological evaluation. The ROI was delimited using rectangular sections from the central region of the alveolus to the middle third of the dental alveolus (1280 µm × 960 µm). %TB meaning percentage of total bone

Microscopic, histometric, and immunohistochemical analyses were performed by a single calibrated examiner who was blinded to the experimental groups (EE). The analyses were conducted using a system composed of a light microscope (Axio Scope^®^, Carl Zeiss Microscopy) with an attached digital camera (AxioCam^®^ MRc5, Carl Zeiss Microscopy) connected to a microcomputer. Photomicrographs of the histological slides were captured using the ZEN 2^®^ software (Blue edition; version 6.1.7601; Carl Zeiss Microscopy). The region of interest (ROI) for the analysis consisted of a rectangle measuring 1280 μm × 960 μm in the central region of the middle third of the dental socket (Figure 1). For histometric analysis of the percentage of bone tissue (PBT), photomicrographs of the histological slides were obtained and analyzed using ImageJ^®^ software (version 1.51i; National Institutes of Health), with the aid of polygon selection tool, which demarcated and measured the area occupied by PBT. The corresponding percentage of the ROI that was occupied by PBT tissue was then calculated.

### Immunohistochemical analysis

For immunohistochemical analyses, antigen retrieval was performed by immersing the histological slides in 10 mM citrate buffer, pH 6.0 (Spring Bioscience), in a pressurized chamber (Decloaking Chamber^®^, Biocare Medical) at 95°C. The histological slides were washed in 0.1 M PBS and pH 7.4. Then, the slides were immersed in a solution consisting of 3% hydrogen peroxide in PBS for 1 hour, and in a solution consisting of 4% skimmed milk powder in PBS for 1 hour to block endogenous peroxidase and biotin, respectively. Blocking of non-specific sites was carried out in 1.5% bovine serum albumin in PBS plus 0.05% Triton^®^ X-100 (Sigma-Aldrich) for 12 hours, as described elsewhere.^36^ The slides were incubated for 24 hours with one of the following primary antibodies: anti-OCN (Abcam Laboratories) and anti-TRAP (Santa Cruz Laboratories). Then, the sections were incubated with biotinylated secondary antibody (Vector Laboratories) for 2 hours and subsequently treated with streptavidin conjugated to horseradish peroxidase (Vector Laboratories) for 2 hours. The 3,3’-diaminobenzidine (Vector Laboratories) was used as a chromogen. The specimens were counterstained with Harris Hematoxylin, then dehydrated in ethanol, cleared in xylene, and covered with mounting medium and glass coverslips. As a negative control, the specimens were subjected to the same procedures described previously, only omitting the use of the primary antibody, as described elsewhere.^37^

For osteocalcin (OCN) analysis, a semi-quantitative analysis was performed respecting the following scoring criteria. Score 0: null immunostaining pattern (total absence of immunoreactive cells (IR) and absence of labeling in the extracellular matrix (ECM); Score 1: low immunostaining pattern (≅ 1/4 of IR cells and weak staining in the ECM); Score 2: moderate pattern of immunostaining (≅ 1/2 of IR cells and moderate staining in the ECM); Score 3: high pattern of immunostaining (≅ 3/4 of IR cells and moderate staining in the ECM). For TRAP analysis, the amount of IR was distributed into the following scores: Score 0: null immunostaining pattern (total absence of IR cells); Score 1: low immunostaining pattern (up to 5 IR cells per field); Score 2: moderate immunostaining pattern (from 6 to 12 IR cells per field); Score 3: high immunostaining pattern (more than 12 IR cells per field).

### Statistical analysis

The Biostat 5.0 (IDSM-Amazonas/Brazil) was used for statistical analysis. For the PBT, one-way analysis of variance (ANOVA) and Tukey’s post-test were used. For the OCN and TRAP immunostaining scores, the Kruskal-Wallis test and the Student-Newman-Keuls post-test were used. Differences were considered significant at p<0.05.

## Results

### Histological analysis

**Figure 2:**
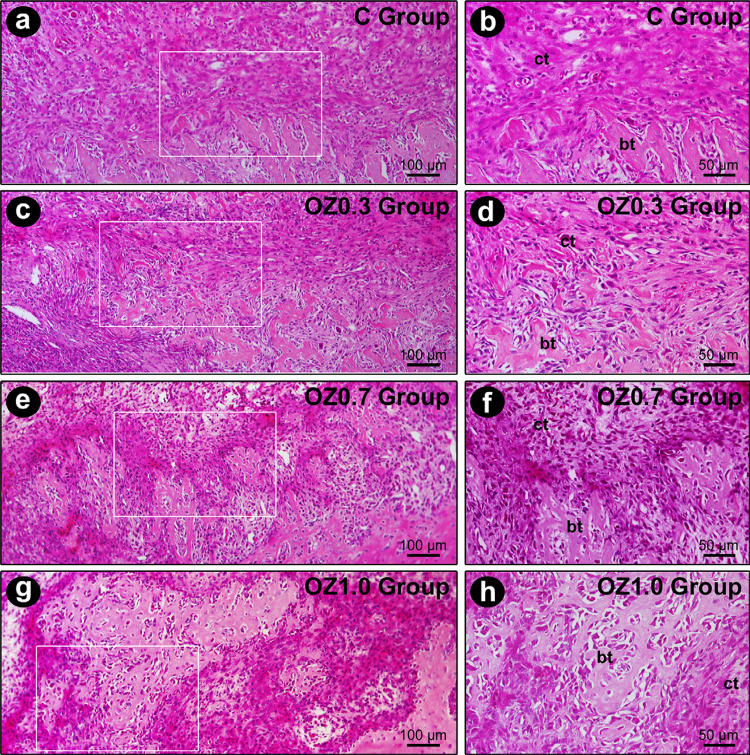
Histological sections of the tooth extraction site seven days postoperatively in the different experimental groups. Photomicrographs showing the histological characteristics of the tissue located in the middle 1/3 of the dental socket in groups C (a-b), OZ0.3 (c-d), OZ0.7 (e-f), and OZ1.0 (g-h), at seven days post-extraction in lower and higher magnifications, respectively. The white squares are the specific zoomed area in the right panel. Abbreviations and symbols: ct, connective tissue; bt, bone tissue. Original magnification: 400×. Scale bars: 50 µm. Staining: Hematoxylin and Eosin (H&E)

**Figure 3:**
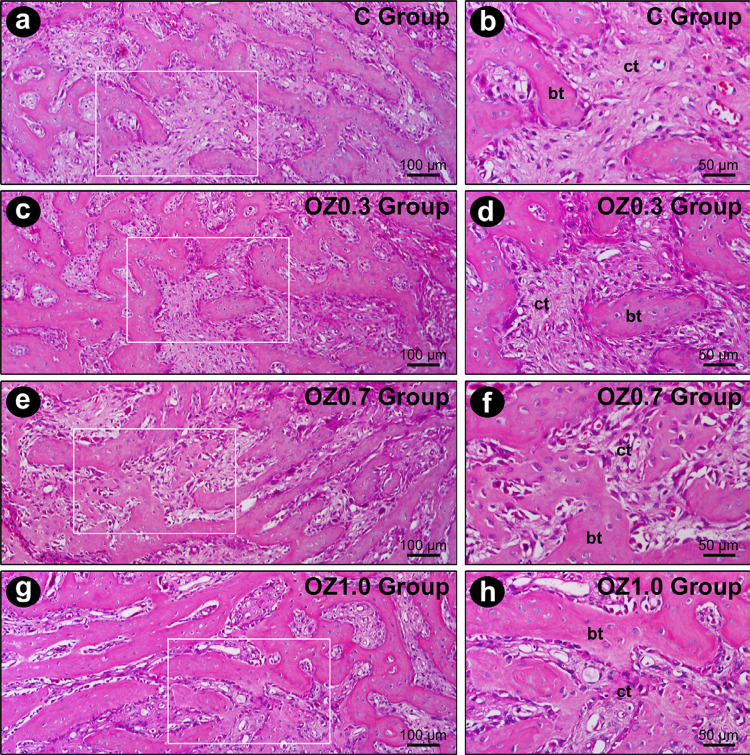
Histological appearance of the tooth extraction site 14 days post-operatively in the different experimental groups. Photomicrographs showing the histological characteristics of the bone tissue located in the middle 1/3 of the dental socket in groups C (a-b), OZ0.3 (c-d), OZ0.7 (e-f), and OZ1.0 (g-h) at 14 days post-extraction in lower and higher magnifications, respectively. The white squares are the specific zoomed area in the right panel. Abbreviations and symbols: ct, connective tissue; bt, bone tissue. Original magnification: 400×. Scale bars: 50 µm. Staining: Hematoxylin and Eosin (H&E)

**Figure 4:**
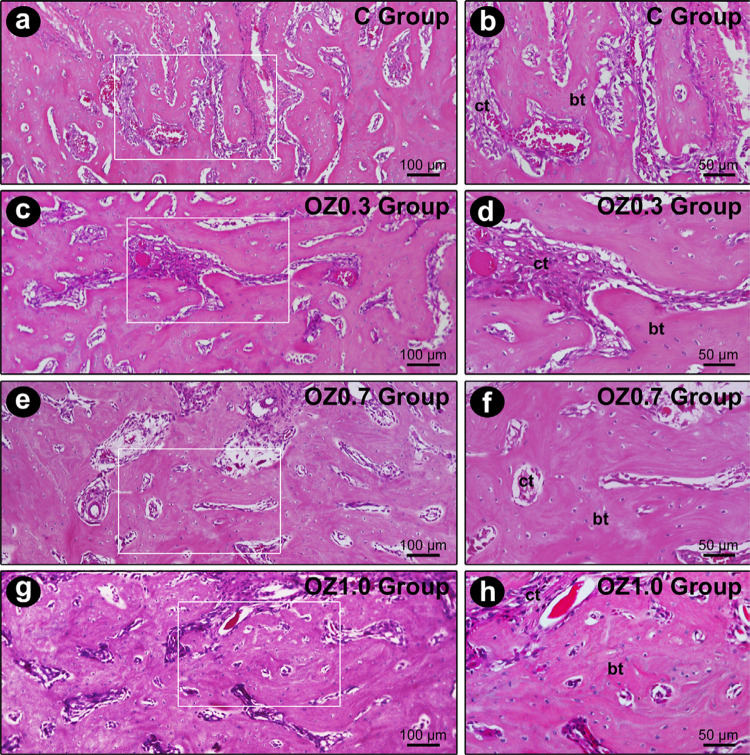
Histological appearance of the tooth extraction site 21 days post-operatively in the different experimental groups. Photomicrographs showing the histological characteristics of the bone tissue located in the middle 1/3 of the dental socket in groups groups C (a-b), OZ0.3 (c-d), OZ0.7 (e-f), and OZ1.0 (g-h) at 21 days post-extraction in lower and higher magnifications, respectively. The white squares are the specific zoomed area in the right panel. Abbreviations and symbols: ct, connective tissue; bt, bone tissue. Original magnification: 400×. Scale bars: 50 µm. Staining: Hematoxylin and Eosin (H&E)

At seven days post-extraction, the dental alveoli were filled with remnants of the blood clot, with highly vascularized and cellularized connective tissue. In the vicinity of the alveolar socket, deposition of bone matrix and formation of fine bone trabeculae were observed, which were more evident in the OZ1.0 Group (Figure 2). At 14 days, a fine network of bone trabeculae, composed of immature bone tissue, occupied a large part of the dental alveoli. These trabeculae were full of osteoblasts with morphological characteristics of intense activity, especially in groups OZ0.7 and OZ1.0, in which the bone trabeculae were evidently denser. Scattered in the bone trabeculae, the connective tissue was highly vascularized and highly cellularized, especially in the OZ0.7 and OZ1.0 groups (Figure 3). At 21 days, a large part of the alveolar socket was occupied by bone trabeculae, whose thickness and level of maturation were greater in the OZ0.7 group, and especially in the OZ1.0 group. The connective tissue located between the trabeculae was highly vascularized and slightly less cellularized than in previous periods (Figure 4). The histological characteristics presented by the OZ1.0 group are consistent with a more accelerated alveolar repair process (Figure 4d).

### Histomorphometric analysis

**Figure 5:**
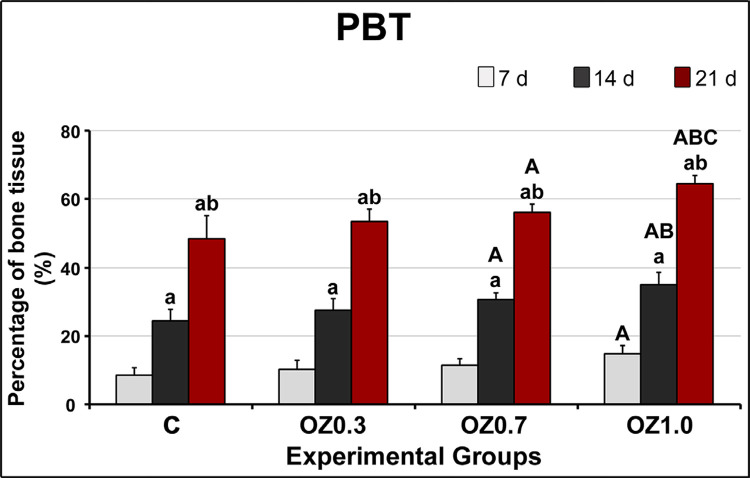
Graph showing the percentage of bone tissue (PBT) in the middle 1/3 of the dental socket in groups C, OZ0.3 (b), OZ0.7 (c), and OZ1.0 at 7, 14, and 21 days after surgery. Symbols: a, difference compared to seven days in the same group; b, difference compared to 14 days in the same group; A, difference compared to group C in the same period; B, difference compared to the OZ0.3 group in the same period; C, difference compared to the OZ0.7 group in the same period. Statistical tests: Shapiro-Wilk Normality Test; Analysis of Variance (ANOVA); Tukey post-test

In the intragroup analysis, the results showed that PBT was higher at 14 days when compared to 7 days in all experimental groups. At 21 days, PBT was increased compared to 7 days and 14 days in all experimental groups (Figure 5). In the intergroup analysis, the OZ0.7 group demonstrated increased PBT when compared to the C group at 14 days and 21 days. In the OZ1.0 group, PBT was higher than group C in all experimental periods. At 14 days and 21 days in the OZ1.0 group, PBT was higher when compared to OZ0.3 group. The PBT at 21 days in the OZ1.0 group was higher than in the OZ0.7 group (Figure 5).

### Immunohistochemical analysis

**Figure 6:**
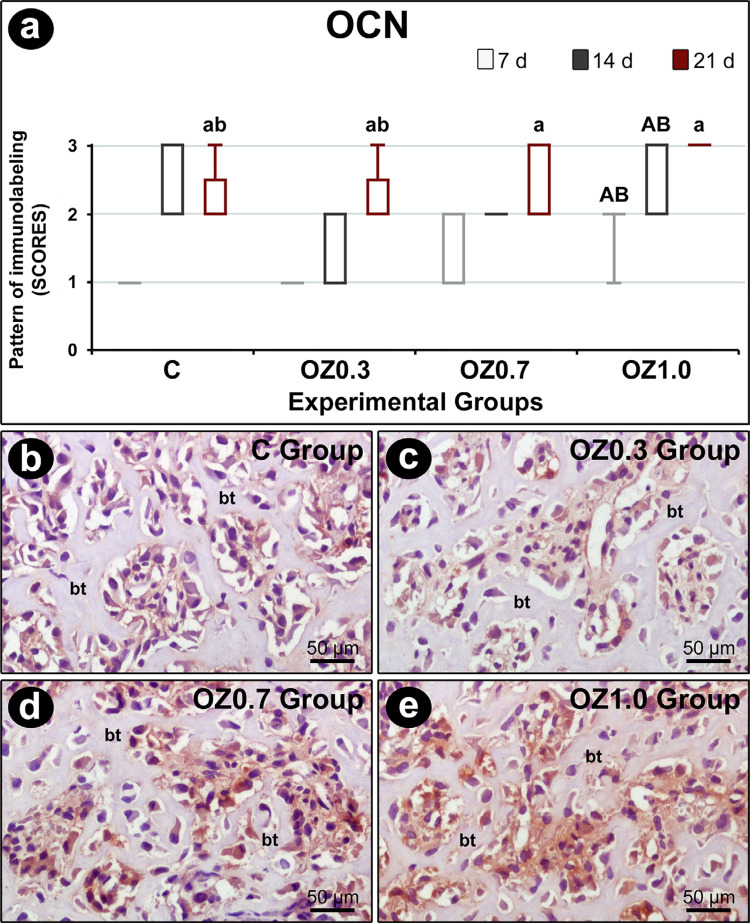
Immunolabeling for OCN at the dental extraction site 21 days post-operatively in the different experimental groups. (a) Graph showing the pattern of immunostaining for OCN in the dental socket in groups C, OZ0.3, OZ0.7, and OZ1.0 at 7, 14, and 21 days after surgery. (b–e) Photomicrographs showing the pattern of immunostaining for OCN in the middle 1/3 of the dental socket in groups C (b), OZ0.3 (c), OZ0.7 (d), and OZ1.0 (e) at 21 post-extraction. Abbreviations and symbols: bt (bone tissue); a, difference compared to seven days in the same group; b, difference compared to 14 days in the same group; A, difference compared to group C in the same period; B, difference compared to the OZ0.3 group in the same period. Original magnification: 1000×. Scale bars: 10 µm. Counterstain: Harris hematoxylin

**Figure 7:**
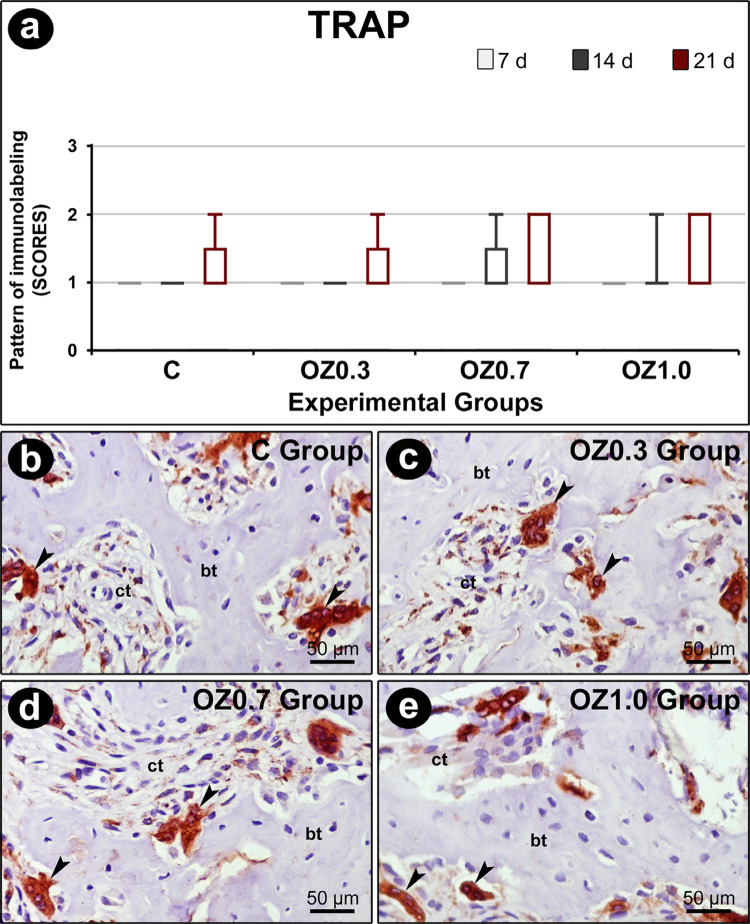
Immunolabeling for TRAP at the dental extraction site at 14 days post-operatively in the different experimental groups. (a) Graph showing the immunostaining pattern for TRAP in the dental socket in groups C, OZ0.3, OZ0.7, and OZ1.0 at 7, 14, and 21 days after surgery. (b–e) Photomicrographs showing the pattern of immunostaining for TRAP in the middle 1/3 of the dental socket in groups C (b), OZ0.3 (c), OZ0.7 (d), and OZ1.0 (e) at 21 days post-extraction. Original magnification: 1000×. Scale bars: 10 µm. Counterstain: Harris hematoxylin. bt: bone tissue; ct: connective tissue

In the intragroup analysis, OCN immunostaining at 21 days was higher than 7 days in all experimental groups. At 21 days in C and OZ0.3 groups, immunostaining for OCN was higher at 21 days compared to 14 days. In the intergroup analysis, the OZ1.0 group at 7 days and 14 days, OCN immunostaining was greater than C and OZ0.3 groups (Figure 6). Both the intragroup analysis and the intergroup analysis found no statistically significant difference in immunostaining for TRAP (Figure 7).

## Discussion

The results of this study demonstrate that systemic treatment with O_3_ positively influenced socket bone repair in rats, characterized by a higher percentage of PBT compared to the control group. Additionally, the data showed that this treatment increased OCN immunostaining, possibly contributing to early bone maturation. However, our findings did not indicate any impact of O_3_ on the reduction of osteoclasts, as evidenced by TRAP-staining. Therefore, our data suggests that the beneficial effect of O_3_ relies on its regenerative properties rather than acting as an antiosteoclastogenic agent. Thus, our results propose that ozone therapy may accelerate the bone repair process in fresh extraction sockets and could be an interesting potential adjunct following dental extractions.

The different dosages of O_3_ used in this study i.e., 0.3mg (15 μg/ml of O_3_), 0.7mg (35 μg/ml of O_3_) and 1.0mg of O_3_/kg (50 μg/ml of O_3_) promoted an increase in PBT, suggesting a positive impact of O_3_ on bone formation. These favorable results were shown to be dose-dependent since the higher increase in PBT was noted in the animals treated with 1.0 mg/kg with a concentration of 50 μg/ml of O_3_ compared to the other doses. These results corroborate previous studies, which demonstrated that low concentrations in dosages of O_3_ (10-80 μg/ml) have been used to promote wound healing and regulate immunity.^7,38^

Ozone therapy is a biological treatment method with a wide range of applications in both medicine and dentistry.^7^ The cascade of compounds derived from O_3_ can act on different targets in the body and under different pathological conditions. Hypotheses regarding its mechanism of action are presented in the literature but are inconclusive and thus should be used cautiously. Studies have shown that O_3_ therapy induces moderate oxidative stress, increases the production of endogenous antioxidants, improves local perfusion and oxygen delivery, and acts on the immune response.^39^ Other studies have reported that O_3_ increases the transport of oxygen through the blood, resulting in an activation of cellular metabolism of aerobic processes (Krebs cycle, glycolysis, β-oxidation of fatty acids) and the use of energy resources. It is also known that the metabolism of inflamed tissues is increased with ozone therapy due to increased oxygenation and reduction of total inflammatory processes, increasing their capacity for tissue regeneration.^12,13,40,41^

Although our animal model and analysis do not allow inference on the exact mechanism of action that resulted in the benefits of treatment with O_3_ in the repair of dental extraction wounds, it can be suggested that these results are due to some potential factors, such as the ability of O_3_ to promote greater blood supply in the injured tissue, increasing its oxygenation. Studies have shown that O_3_ can improve oxygen metabolism, stimulating important enzymes that participate in its metabolism, increasing oxygen saturation in circulating blood with a consequent increase in oxygen supply to the body cells.^42^ Moreover, the bactericidal action of ozone is based on its robust oxidation properties with the concomitant formation of free radicals, which favor the eradication of almost all microorganisms.^14,38^

The literature presents no consensus regarding the most effective dose of O_3_ for improving the repair of bone wounds and tissue regeneration. Studies have shown that a dose of 0.7 mg/kg administered intraperitoneally benefited long-term repair of tooth extraction wounds, as well as that systemic O_3_ application could accelerate alveolar bone healing after tooth extraction.^12^ In the treatment of induced periodontal disease, ozone therapy was suggested to reduce osteoclastic activity and alveolar bone loss.^43^ The current findings did not result in any effect of ozone therapy in the number of positive osteoclasts, which differs from the study of Saglam, et al.^43^ (2020). The differences in osteoclastic activity between these studies might be accounted for the different route of administration of ozone (topically vs systemically), animal model employed (tooth extraction vs periodontitis), different doses, and number of injections performed (single injection vs multiple).

We highlight some limitations of this study. Firstly, we did not evaluate the toxicity of systemic administration of ozone in our animals. Thus, further studies should accomplish the hematoxicity test to provide insights regarding its side effects. Secondly, the systemic administration of ozone does not guarantee the same concentration at the oral tissue, which was also not investigated in our study. Thus, further studies using local treatment should be conducted to achieve more reliable local effects. Finally, the animal model employed can be optimized using extraction of molar teeth instead of central incisor. There is a lack of studies on the effects of local ozone therapy on the repair process, thus we suggest further studies to explore the biological mechanisms involved, including possible influences on other adjuvant therapeutic modalities, relevant to the repair process. Studies involving different dosages, therapeutic protocols, and routes of administration that enhance its biological effects and increase the bioavailability of the ozone in affected tissues are necessary so that clinical trials can be considered.

## Conclusions

Within the limitations of this *in vivo* study, our data suggest that ozone therapy can benefit bone repair and increase the newly formed bone in the alveolar socket following tooth extractions in rats.
